# Experimental and numerical study on the flexural capacity of fiber mesh fabric-reinforced RC slabs

**DOI:** 10.1371/journal.pone.0348872

**Published:** 2026-05-18

**Authors:** Chunxiang Guo, Xiaoming Bao, Zhengge Shao, Shuai Yan, Xiangcheng Zhang, Chang Ge, Zhichun Wu, Ke Jiang

**Affiliations:** 1 4th Construction Co., Ltd. of China Construction 5th Engineering Bureau, Luoyang, China; 2 School of Civil Engineering, Zhengzhou University, Zhengzhou, China; 3 Zhengzhou University Industrial Technology Research Institute Co., Ltd, Zhengzhou, China; 4 School of Mechanics and Safety Engineering, Zhengzhou University, Zhengzhou, China; University of Vigo, SPAIN

## Abstract

This study experimentally investigates the effects of embedded carbon and glass fiber mesh fabrics on the flexural performance of one-way reinforced concrete (RC) slabs. Five slab specimens, incorporating different fiber types (carbon and glass) and areal densities (120 g/m^2^ and 240 g/m^2^), were subjected to four-point bending tests. The experimental results demonstrate that fiber mesh reinforcement significantly enhances the mechanical behavior of RC slabs, particularly in terms of cracking control and load-bearing capacity. In terms of crack development, fiber mesh fabrics-reinforced slabs exhibited denser and finer crack patterns, with the 240 g/m^2^ carbon fiber mesh showing the narrowest crack widths and most effective suppression of crack propagation. Compared to the unreinforced control specimen, the cracking load of fiber mesh fabrics-reinforced slabs increased by 14.2% to 114.2%, yield load by 21.3% to 48.7%, and ultimate load by 1.4% to 21.4%. Among them, the specimen reinforced with 240 g/m^2^ carbon fiber mesh exhibited the most substantial improvement, achieving 114.2% higher cracking load, 48.7% higher yield load, and 21.4% higher ultimate load. At the same areal density, carbon fiber mesh outperformed glass fiber mesh, providing up to 25% improvement in cracking load, 18.8% in yield load, and 16.6% in ultimate load. Increasing the areal density from 120 g/m^2^ to 240 g/m^2^ also enhanced flexural performance, with cracking load improvements of 50% observed for both glass and carbon fiber mesh fabrics-reinforced slabs. Finally, the finite element (FE) models of the slabs were developed using the ABAQUS software. The FE simulations accurately replicated the crack development, deflection behavior, and load-transfer mechanisms observed in the experiments, validating the model’s reliability. These results provide critical insights for optimizing the design of fiber-reinforced concrete slabs.

## 1 Introduction

Reinforced concrete (RC) slabs are essential structural elements widely used in buildings, bridges, tunnels, and industrial constructions. Serving as primary load-bearing components, slabs play a critical role in distributing loads and ensuring structural integrity [1]. Due to their large surface areas combined with relatively small thicknesses, slabs are prone to various types of cracks during construction and service stages, mainly caused by thermal stresses, shrinkage, and external loading [2,3]. These cracks often lead to issues such as leakage, reinforcement corrosion [4,5], and degradation of structural performance [6], which ultimately compromise the durability and safety of concrete structures [7].

To improve the flexural performance of concrete slabs, multiple approaches have been explored [8]. Increasing the steel reinforcement ratio is a conventional measure, but it often results in congestion, increased construction complexity, and cost. Incorporating discrete fibers like steel, polypropylene, or basalt fibers into concrete enhances toughness and ductility but has limited effect on initial cracking resistance [9]. Prestressed concrete technology effectively controls cracking but entails complex processes, higher costs, and potential prestress loss over time [10]. These drawbacks restrict its broad application in many practical engineering contexts. Recently, high-performance materials have been increasingly utilized to enhance the flexural capacity of RC slabs. Among them, fiber-reinforced polymer (FRP) strengthening systems have been widely adopted due to their superior specific strength, lightweight nature, and corrosion resistance [11–13]. For example, Sharaky *et al.* [14] investigated the flexural performance of one-way RC slabs strengthened with externally bonded (EB) and near-surface mounted (NSM) CFRP techniques revealed that NSM strengthening outperformed EB, increasing load-bearing capacity by up to 264.5%. While expanding the strengthening area effectively enhanced load capacity, the potential for end shear failure required careful consideration. Finite element analysis further confirmed the influence patterns of key parameters on strengthening effectiveness. Correia *et al.* [15] investigated 12 CFRP-strengthened concrete slabs revealed that the 0.4% prestress level with mechanical anchorage and sandblasted surface treatment achieved optimal flexural performance, outperforming other combinations of anchorage types, prestress levels, laminate dimensions, and surface preparation methods. Attia *et al.* [16] tested twelve one-way slabs reinforced with BFRP or GFRP bars in basalt macro-fiber concrete evaluated the effects of FRP bar type, reinforcement ratio ranging from 1.4 to 2.8 times the balanced ratio, and BMF content up to 2 percent by volume. The results showed basalt macro-fibers significantly improved both ductility and load-bearing capacity while demonstrating that current design codes can effectively predict the structural performance, thereby confirming BMF’s potential as a sustainable alternative to conventional reinforcing fibers. Al-Hamrani *et al.* [17] investigated eight one-way slabs reinforced with BFRP bars through four-point bending tests revealed that increasing the reinforcement ratio enhanced shear capacity by 25–29%, while adding 0.75% basalt macro fibers (BMF) provided additional improvement – though less pronounced at higher reinforcement ratios, with the proposed analytical model demonstrating excellent accuracy. However, the long-term durability of conventional FRP sheet composites remains a concern, particularly under harsh environmental exposures, which may compromise their effectiveness over service life [18,19]. In addition, FRP rebars have low shear strength, making them less effective in resisting shear stresses. Moreover, their bond with conventional concrete is insufficient, leading to potential slippage or bond failure, which does not improve the cracking behavior of the slab.

To overcome this, the combination of FRP fabrics with cementitious matrices has gained significant attention for improving the flexural performance of RC slabs, considering the durability problem. FRP meshes such as glass, carbon, or basalt fibers are embedded in a cement-based matrix, offer improved crack control, enhanced load-bearing capacity, and better durability without a considerable increase in structural weight or complexity (it is also called textile-reinforced concrete [20]). In addition to these mechanical performance advantages, the application of fiber mesh fabrics also offers significant ecological and economic benefits, aligning with the modern construction industry’s sustainability requirements [21,22]. D. Erten et al. [23] demonstrated that replacing traditional reinforced concrete structures with mesh fabrics can significantly reduce greenhouse gas emissions and material energy consumption. They compared the environmental and economic benefits of different structural systems, emphasizing that the use of mesh fabrics provides a comprehensive cost-performance advantage. Fifer-Bizjak, K. et al. [24] found that geosynthetics are more sustainable and economical than traditional concrete structures under most environmental indicators. They particularly show improvements in material usage, maintenance requirements, and long-term life-cycle impacts. Several experimental investigations have demonstrated that RC components reinforced with such composite systems exhibit marked improvements in cracking load, yield load, and ultimate strength. For example, Abd et al. [25] performed four-point bending tests on one-way slabs strengthened with carbon fiber mesh fabrics in both single-layer and double-layer configurations. The results showed significant improvements in cracking load, yield load, and ultimate load compared to unreinforced slabs. Similarly, Kim *et al*. [26,27] reported a 24% increase in flexural bearing capacity for slabs reinforced with prefabricated, spliced carbon fiber mesh fabrics. Akbari *et al*. [28] investigated multiple parameters (including fabric type, number of layers, and reinforcement ratio) on RC beam performance. Their findings confirmed that increasing the number of carbon fiber mesh fabric layers substantially enhanced the ultimate load capacity, crack control, and ductility. In terms of shear strengthening, Hamoda *et al*. [29] introduced a hybrid system combining strain-hardening cementitious composites (SHCC) with embedded glass fiber mesh fabric, applied by spraying or casting. This approach led to significant improvements in shear capacity, crack distribution, and ductility, shifting the failure mode from brittle shear to ductile flexure. Aylin *et al*. [30] evaluated the strengthening of self-compacting concrete (SCC) beams with layered glass fiber mesh fabrics. Their results demonstrated enhanced ductility and flexural strength, although performance degradation was observed under sodium sulfate corrosion, highlighting the durability challenge. A review of the aforementioned studies reveals that most existing experimental research has primarily focused on the mechanical performance of retrofitted concrete members, in which fiber mesh fabrics are externally bonded to the surface of existing structures using mortar or adhesive materials. These approaches do not incorporate the mesh as an integral high-performance reinforcement during the initial casting stage. As a result, they fail to fully capture the role of fiber mesh fabric during the early service stages of the structure, such as initial cracking and pre-yield deformation behavior. This limitation restricts a deeper understanding of the reinforcement mechanism and hinders the optimization of the structural performance provided by the embedded fiber mesh fabric.

Furthermore, it is crucial to recognize the limitations within current international codes of practice regarding such composite systems. Conventional structural design standards, such as ACI 318 and the CEB-FIP Model Code, are primarily formulated for traditional steel or discrete FRP bar reinforcements. Although specific guidelines exist for externally bonded strengthening systems (e.g., ACI 549), there remains a significant lack of standardized predictive models and design provisions for continuous fiber meshes integrally embedded during the initial casting stage. This limitation in current codes further highlights the necessity for systematic experimental investigations to establish reliable design methodologies [31,32]. To fill this research gap, the present study adopts an embedded fiber mesh reinforcement strategy for concrete slabs, in which fiber mesh is placed at the bottom of the RC slab before steel reinforcement and concrete casting, and cast simultaneously with the RC slab. Five specimens were designed and fabricated using fiber meshes of different materials (glass fiber and carbon fiber) and surface densities to investigate their effects on structural performance. Through experimental testing, the failure modes, load–deflection responses, load-carrying capacities, and material strain distributions of the specimens were analyzed. The effects of fiber mesh type and surface density on the crack resistance, bending stiffness, and bending load-carrying capacity of RC slabs were systematically evaluated. Furthermore, a finite element model was developed using ABAQUS to simulate the flexural performance of fiber mesh-reinforced slabs. The findings of this study aim to provide a deeper understanding of the role of fiber mesh fabrics in enhancing crack resistance and flexural capacity of RC slabs.

## 2 Experimental program

### 2.1 Specimen design

**Fig 1:**
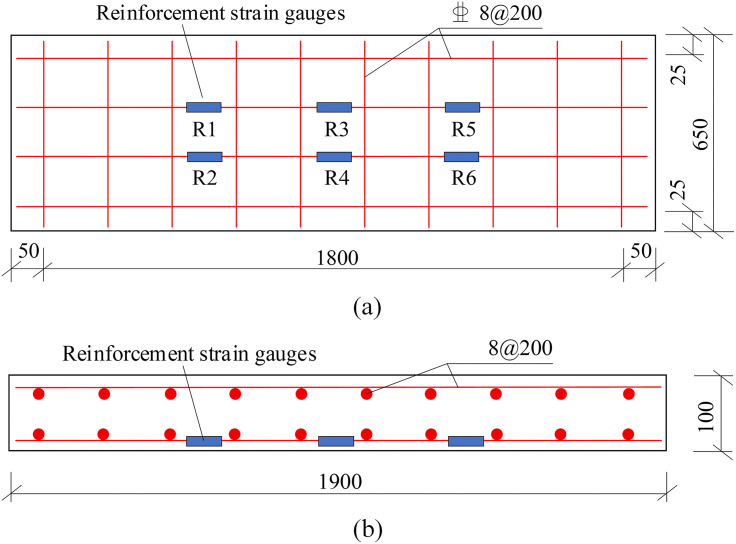
Reinforcement diagram of the specimen (unit: mm). **(a)** Plan view. **(b)** Side view.

Five reinforced concrete slab specimens with identical dimensions and reinforcement but varying types and weights of fiber mesh fabrics were fabricated [33,34]. The dimensions were specified as 1900mm × 650 mm × 100 mm. The reinforcement details of the specimens are illustrated in Fig 1, while the specimen numbering and design parameters are tabulated in Table 1. The concrete slabs were designed with an orthogonal double-layered reinforcement mesh, employing 8 mm-diameter steel bars spaced at 200 mm intervals in both directions, and a concrete cover thickness of 15 mm was maintained. Within this reinforcement layout, the longitudinal steel bars are placed on the outer side of the transverse steel bars. The longitudinal steel bars are designated as the primary flexural reinforcement to resist the applied bending moments. The transverse steel bars function solely as distribution and temperature/shrinkage reinforcement, in accordance with standard design provisions, to mitigate secondary stresses and maintain the spatial stability of the reinforcement cage during casting. Among the five specimens, specimen DB-1 served as the control group, with no fiber mesh fabric applied to its soffit. The soffits of specimens GDB-2 and GDB-3 were reinforced with glass fiber mesh fabrics weighing 120g/m^2^ and 240g/m^2^, respectively. Similarly, the soffits of specimens CDB-4 and CDB-5 were strengthened with carbon fiber mesh fabrics of 120g/m^2^ and 240g/m^2^ respectively. The specimen fabrication process was carried out in the following steps: setting up the formwork, tying the reinforcement cage, laying the fiber mesh, applying the strain gauges to the reinforcement, and finally, pouring the concrete.

**Table 1:** Specimen design variables.

Specimen No.	Fiber mesh fabric type	Specifications
DB-1	/	/
GDB-2	Glass	120g/m^2^
GDB-3	Glass	240g/m^2^
CDB-4	Carbon	120g/m^2^
CDB-5	Carbon	240g/m^2^

### 2.2 Materials

**Fig 2:**
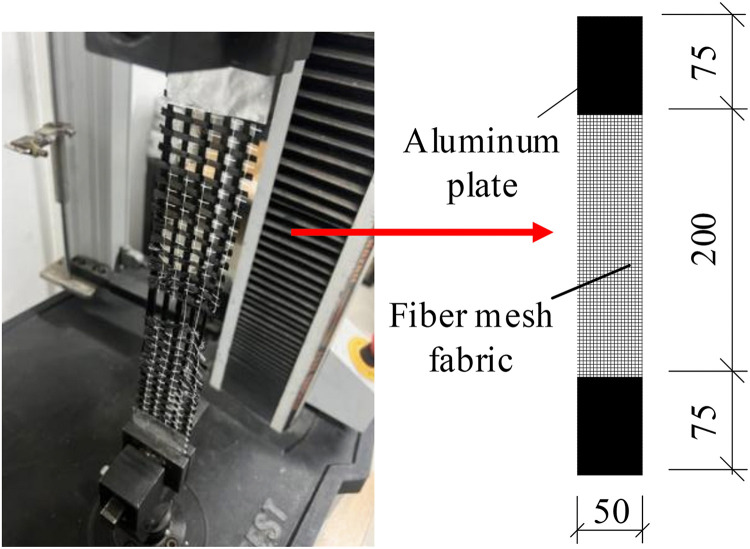
Tensile testing of fiber mesh fabric.


The specimens were fabricated using C30 concrete and HRB400 steel reinforcement. During the casting process, three 100 mm concrete cube specimens were simultaneously cast and cured under identical conditions. Compressive tests were conducted using a YES-3000 digital compression testing machine. By applying a size conversion factor of 0.95 in accordance with the GB/T 50107−2010 [35] standard, the average compressive strength of concrete was determined to be 33.4 MPa with an elastic modulus of 30.8 GPa. Similarly, three standard tensile coupons were tested for the steel reinforcement. The steel reinforcement exhibited an average yield strength of 418 MPa, tensile strength of 613 MPa and elastic modulus of 200 GPa, as measured following the GB/T 228.1−2021 “*Metallic materials – Tensile testing-Part 1: Method of test at room temperatur*e” [36]. The fiber mesh fabric specimens were prepared according to the GB/T 7689.5−2013 “*Reinforcements – Test methods for woven fabrics*” [37], with the configuration illustrated in Fig 2. The fiber mesh fabric was cut into 350 mm × 50 mm strips which were then folded along their longitudinal centerline and secured with 150 mm × 50 mm aluminum clamping plates. Both ends were encapsulated with resin adhesive, maintaining an effective length of 200 mm. For each fiber mesh configuration and testing direction, five replicate specimens were prepared and tested to ensure data reliability. The cross-sectional area of fiber bundles was calculated using Equation (1). The basic material property parameters are presented in Table 2.

**Table 2:** The basic material property parameters of fiber mesh fabric.

Fiber mesh fabric type	Fiber bundle weight （g/km）	Fiber bundle density （g/cm^3^）	Cross-section area （mm^2^）
Glass (120g/m^2^, 240g/m^2^)	330	2.54	0.13
Carbon (120g/m^2^, 240g/m^2^)	198	1.8	0.11

$$\begin{array}{c} A_{f}=\frac{T_{ex}}{D_{f}} \end{array}$$ (1)

where *A*_f_ is the cross-sectional area of individual fiber bundles; *T*_ex_ is the linear density (mass per 1000m length), and *D*_f_ is the fiber bundle density.

**Fig 3:**
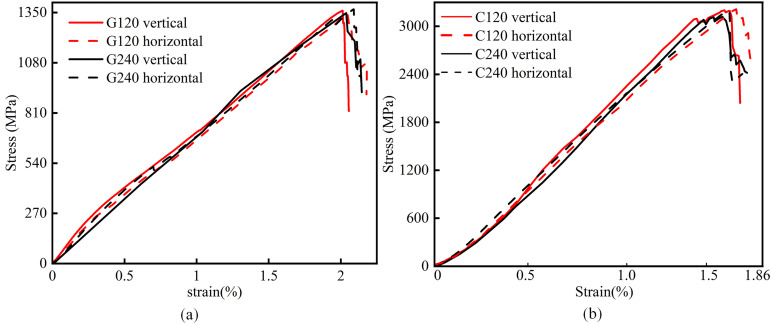
Stress-strain curve for fiber mesh fabrics. **(a)** Glass fiber mesh fabric. **(b)** Carbon fiber mesh fabric.

Tensile tests were conducted on fiber mesh fabric specimens following the GB/T 3354–2014 “*Test method for tensile properties of oriented fiber reinforced polymer matrix composite materials*” [38]. The stress-strain curves for different fiber mesh fabrics in both longitudinal and transverse directions are presented in Fig 3, with average characteristic data summarized in Table 3. The experimental results demonstrate that the tensile behavior of glass fiber mesh fabric can be characterized by two distinct phases: elastic phase and descending phase. During the elastic phase, the fiber bundles were uniformly stressed, maintaining a constant curve slope until fracture occurred at approximately 1380 MPa, corresponding to 2.0% elongation. Subsequently, the stress decreased rapidly in the descending phase. In contrast, carbon fiber mesh fabric exhibited three-phase behavior: initial nonlinear phase, elastic phase, and descending phase. The initial phase showed progressively increasing slope as fiber bundles became gradually stressed. Upon reaching 100 MPa, the elastic phase commenced. Premature fiber bundle fractures caused slight stress reductions before peak strength (3000–3300 MPa) was attained at 1.62% elongation. The descending phase displayed local fluctuations due to residual strength retention in partially fractured bundles.

**Table 3:** Mechanical properties of fiber mesh fabric.

Fiber mesh fabric type	Direction	Tensile strength	Cross-section area	Breaking elongation	Elastic modulus
		(MPa)	(mm^2^)	(%)	(GPa)
Glass (120g/m^2^)	vertical	1386.6	1.54	2.04	67.5
	horizontal	1373.7	0.91	2.02	68.2
Glass (240g/m^2^)	vertical	1382.4	1.82	2.0	69.1
	horizontal	1380.7	1.54	1.99	68.8
Carbon (120g/m^2^)	vertical	3035.6	0.88	1.65	197.3
	horizontal	3163.4	0.88	1.63	192.4
Carbon (240g/m^2^）	vertical	3296.2	1.54	1.62	203.5
	horizontal	3269.2	1.21	1.62	198.0

### 2.3 Measurement plan

The strain measurement system was carefully designed to capture the mechanical behavior of the structural components. Foil-type electrical resistance strain gauges (Model BF120−3AA) were strategically positioned at three critical locations along the lower tensile reinforcement bars: the quarter-span (1/4), mid-span (1/2), and three-quarter-span (3/4) positions. This configuration enables monitoring of strain distribution and potential moment redistribution along the span length. For concrete strain measurement, five strain gauges with a larger gauge length (Model Bx120−50AA) were systematically installed at the mid-span section with the following arrangement: one at the bottom surface (maximum tensile zone), one at the top surface (compression zone), and three along the side surface spaced at equal intervals through the slab thickness (100 mm). This vertical array allows for comprehensive assessment of strain gradients and neutral axis development during loading. Displacement monitoring was implemented using 50 mm-range linear variable differential transformers (LVDTs) positioned at the bottom soffit at the quarter-span (D2), mid-span (D3), and three-quarter-span (D4) locations. Additionally, two LVDTs (U1, U5) were positioned at the top supports to monitor potential settlement. The instrumentation scheme was designed to capture all essential structural responses while maintaining measurement redundancy for data reliability.

### 2.4 Test setup and loading protocol

**Fig 4:**
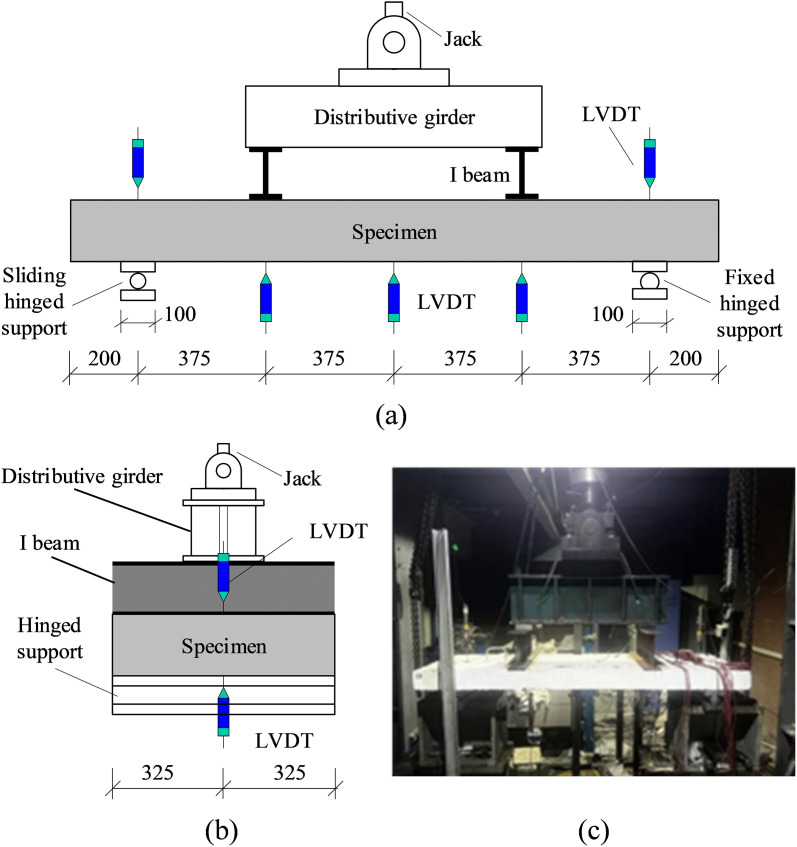
Test setup for RC slab specimens. **(a)** Front view diagram. **(b)** Side view diagram. **(c)** Field loading diagram.

The experimental setup is illustrated in Fig 4. The system consists of rigid distribution beams, I-beams, steel bearing plates, displacement transducers, and hinged supports. A four-point bending test configuration was adopted to simulate uniformly distributed loading conditions on the one-way slab specimens, with support conditions and quasi-static loading procedures established in strict compliance with the GB/T 50152−2012 “*Standard test methods for concrete structures*” [39]. The support system was designed with an asymmetric arrangement featuring a sliding hinged support at the left end and a fixed hinged support at the right end. The centerlines of the hinged supports were positioned 200 mm from the specimen ends, resulting in a clear span of 1500 mm for the one-way slabs. Vertical loading was applied through a hydraulic jack and transferred to the top surface of the specimens via a distribution beam and I-beam assembly. To prevent localized bearing failure, a layer of fine sand was uniformly distributed between the I-beam and the slab surface to facilitate stress distribution. The centroidal axis of the I-beam section (i.e., the loading point) was located at the quarter-point of the specimen’s clear span. It should be noted that the two longitudinal edges of the slabs were completely free without any torsional or vertical restraint. Furthermore, the I-beams applied a uniform line load across the entire 650 mm width of the specimens. This combination of strictly two-sided support and uniform transverse load introduction ensured that the structural response was governed entirely by one-way cylindrical bending rather than two-way plate action.

The loading protocol commenced with a preloading phase prior to formal loading. This preliminary stage served to verify the stability of both the actuator and support system through observational data, while simultaneously confirming the proper functioning of all strain gauges and displacement transducers. The quasi-static test was conducted under strict load control. Before cracking, the load increments were 2.5 kN per level. After cracking, the load increments were 5 kN per level. Following each loading increment, a 10-minute holding period was systematically maintained to allow for comprehensive

observation, during which maximum crack widths were precisely recorded using a crack microscope and crack propagation patterns were carefully documented to monitor damage progression.

## 3 Test results and analysis

### 3.1 Test process and failure pattern

**Fig 5:**
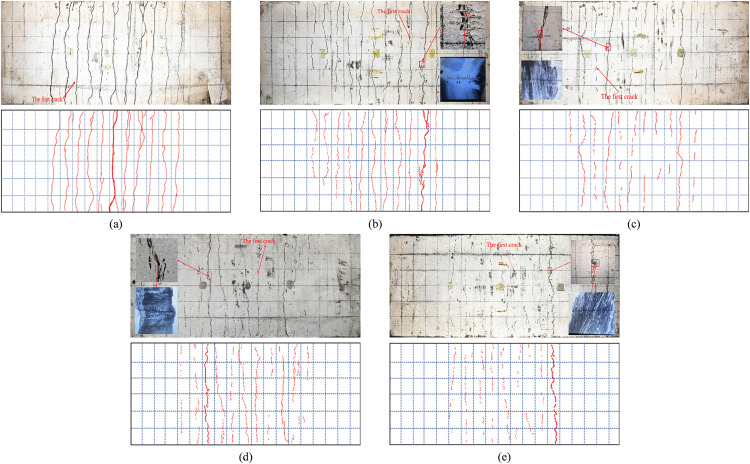
Ultimate failure pattern of slab specimens. **(a)** DB-1. **(b)** GDB-2. **(c)** GDB-3. **(d)** CDB-4. **(e)** CDB-5.

All specimens exhibited characteristic flexural failure modes. Initial cracking was observed in the control specimen DB-1 when the load reached 17.5 kN, with the first crack appearing adjacent to the left loading point and measuring 0.18 mm in width. Progressive crack formation was induced in the midspan region with increasing load. The test was terminated at a midspan deflection of 30 mm, revealing 11 fully penetrating cracks within the loading points, among which the primary crack was localized at midspan, as illustrated in Fig 5(a).

Distinct cracking characteristics were demonstrated by the glass fiber-reinforced specimens, as shown in Fig 5(b) and 5(c). For specimen GDB-2 with 120 g/m^2^ fiber mesh fabrics, cracking was initiated at 20 kN, manifested as a 250 mm non-penetrating crack (0.06 mm width) near the right loading point. Specimen GDB-3 with 240 g/m^2^ fiber mesh fabrics developed three discrete cracks (40−60 mm length, 0.08 mm width) near the left loading point at 30 kN. Concrete spalling and bond failure were observed in both specimens between 45−50 kN. Upon termination (30 mm deflection), GDB-2 was found to contain 11 cracks (including 7 penetrating cracks) with primary cracking near the right loading point, while GDB-3 developed 11 cracks (3 penetrating cracks) with main crack formation between the left loading point and midspan region.

Superior crack resistance was displayed by the carbon fiber-reinforced specimens CDB-4 and CDB-5, as presented in Fig 5(d) and 5(e). The failure process was characterized as follows: Five discontinuous cracks (50−200 mm length, 0.08 mm width) were formed near midspan in CDB-4 (120 g/m^2^) at 25 kN, whereas multiple microcracks (<30 mm length, 0.06 mm width) were developed in CDB-5 (240 g/m^2^) at 37.5 kN. Crack coalescence was observed in CDB-4 during subsequent loading, while stable microcrack distribution was maintained in CDB-5. When the maximum crack width reached 1.5 mm, 12 cracks (6 penetrating cracks) were recorded in CDB-4 with primary cracking near the left loading point, compared to only 9 cracks (1 penetrating crack) with narrow distribution in CDB-5.

Comparing the five specimens, fiber rupture occurred in GDB-2, GDB-3, and CDB-4, whereas fiber bundles remained intact in CDB-5. Fiber reinforcement effectively constrained crack development, transforming the continuous penetrating cracks seen in control specimen DB-1 into discrete, densely distributed patterns. Higher areal density meshes demonstrated enhanced suppression, particularly in limiting crack length. Specific failure mechanisms were diagnosed through combined visual, acoustic, and microscopic evidence. Mesh rupture was identified by intermittent snapping sounds during the post-yielding stage and confirmed via crack microscopy, which revealed fractured filaments within primary cracks (see magnified insets in Fig 5b-5e). Debonding and concrete cover separation were characterized by localized spalling of the concrete soffit and loss of interfacial adhesion, a phenomenon most pronounced in glass fiber specimens GDB-2 and GDB-3 between 45 kN and 50 kN. These bond failures prematurely limited the reinforcement’s load-sharing efficiency.

### 3.2 Crack width

**Fig 6:**
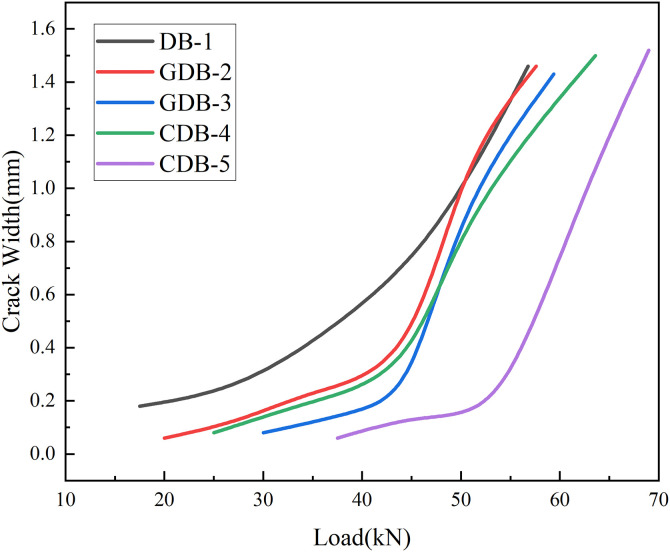
Load-maximum crack width curves.

The comparative analysis of maximum crack width development across all five specimens is graphically presented as load versus maximum crack width curves in Fig 6. As shown in the figure, during the initial loading phase below 40 kN, a relatively gradual progression of crack width expansion was observed in all specimens, exhibiting an approximately linear growth pattern. This stage was characterized by stable crack propagation behavior without significant width acceleration.

A distinct behavioral transition was identified within the 45 ~ 50 kN loading range. For the control specimen DB-1, which lacked fiber reinforcement, the maximum crack width was consistently recorded within the constant-moment region (pure bending zone) near the midspan. In this region, DB-1 demonstrated rapid crack width acceleration, a phenomenon attributed to the complete transfer of tensile stresses to the bottom reinforcement. Conversely, for the glass fiber-reinforced specimens GDB-2 and GDB-3, the governing maximum cracks shifted towards the shear-flexure zone near the loading points. The sudden crack width increases detected in these specific regions between 45 kN and 50 kN directly corresponded to the local debonding and concrete cover spalling phenomena described previously. These rapid increases in crack width primarily resulted from the glass fiber’s relatively low elastic modulus, combined with the progressive bond failure and substantial plastic deformation of the steel reinforcement.

In contrast, carbon fiber-reinforced specimens CDB-4 and CDB-5 exhibited superior performance characteristics. The enhanced tensile strength and elastic modulus of the carbon fiber mesh were found to effectively improve flexural stiffness, leading to reduced midspan deflections under equivalent loading conditions. This stiffness enhancement effect delayed the onset of plastic deformation in the bottom reinforcement, consequently producing more gradual crack width development curves. Furthermore, the carbon fiber reinforcement system promoted more uniform stress distribution, enabling stable crack width progression to be maintained up to the 55 kN loading level.

In addition, Chinese codes usually control crack width according to environmental class, with common limits of 0.40 mm, 0.30 mm, 0.20 mm, and 0.15 mm. In Eurocode 2 [40], the most typical control value is 0.30 mm. ACI 224R recommends about 0.41 mm for general indoor dry environments, about 0.30 mm for humid environments, about 0.18 mm for environments exposed to deicing salts or corrosion, about 0.15 mm for seawater or severe exposure conditions, and about 0.10 mm for water-retaining structures. Under the experimental conditions of this study and the normal service load of 40 kN, the maximum crack widths of the CFRP-strengthened specimens (CDB-4 and CDB-5) were about 0.25 mm and 0.1 mm, respectively. These values were lower than the above code limits. This comparison confirms that the embedded fiber mesh effectively satisfies international serviceability requirements.

### 3.3 Load-deflection curves

**Fig 7:**
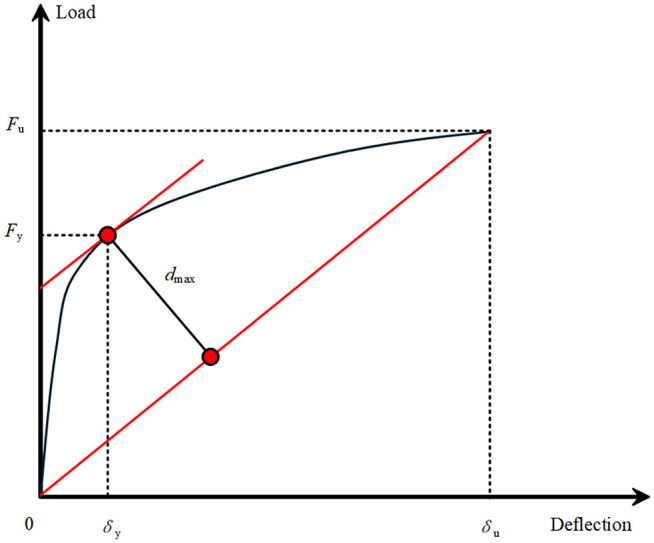
“Farthest Point” method schematic‌‌ diagram.

Based on the loading process of the specimens, the structural response can be divided into three characteristic stages represented by distinct points: cracking point, yielding point, and ultimate point, with their corresponding loads defined as cracking load *F*_*cr*_, yield load *F*_*y*_, and ultimate load *F*_*u*_, respectively. The cracking load is defined as the load value when the first crack appears in the bending region of the specimen. The yield load is determined using the “farthest point” method proposed in reference. Since flexural members have clearly defined stress states in each section, the point on the load-displacement curve that is farthest from the straight line connecting the origin and the peak point is identified as the yield point (as shown in Fig 7), and its corresponding load is taken as the yield load. The ultimate load is defined as the load at structural failure. The specimen is considered to have failed when any of the following conditions is met during loading: 1) The maximum crack width in the bending region reaches 1.5 mm; 2) The midspan deflection reaches 1/50 of the net span (i.e., 30 mm); 3) The tensile reinforcement at the bottom of the slab fractures or reaches a maximum strain of 0.01; 4) The concrete at the top of the slab cracks or crushes under compression. To ensure the comparability of the ultimate load-bearing capacity across different specimens, it is essential to identify the specific governing criterion for each test. Table 4 maps each specimen to the exact condition that triggered the termination of loading. For specimens with higher ductility (DB-1, GDB-2, and GDB-3), the ultimate state was defined by the midspan deflection limit (Condition 2). For specimens with higher stiffness (CDB-4 and CDB-5), the maximum crack width reached the 1.5 mm limit (Condition 1) before the deflection threshold, thus governing the termination.

**Table 4:** Governing ultimate state criteria for each specimen.

Specimen	Primary Failure Characteristic	Governing Stopping Condition
DB-1	Steel yielding and large deflection	Condition (2): Midspan deflection reached 30 mm
GDB-2	Flexural yielding with partial debonding	Condition (2): Midspan deflection reached 30 mm
GDB-3	Flexural yielding with fiber rupture	Condition (2): Midspan deflection reached 30 mm
CDB-4	High stiffness with localized wide cracks	Condition (1): Maximum crack width reached 1.5 mm
CDB-5	High stiffness with multiple cracking	Condition (1): Maximum crack width reached 1.5 mm

**Fig 8:**
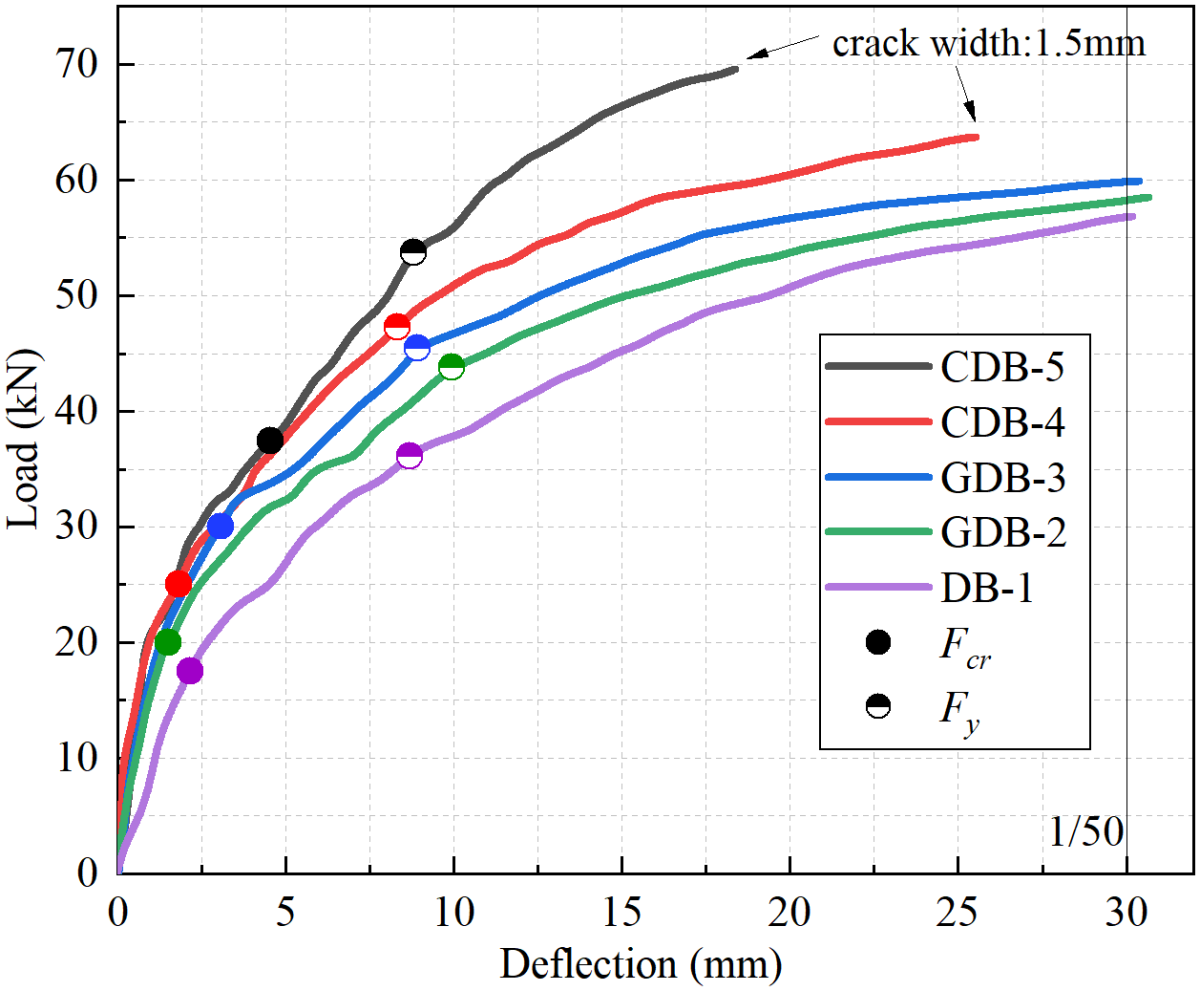
Load-deflection curves at mid-span.

The load-midspan deflection relationships for all five specimens are presented in Fig 8. Prior to cracking, an approximately linear correlation between load and deflection was observed. The slopes of the load-deflection curves were found to be steeper for specimens GDB-2, GDB-3, CDB-4 and CDB-5 compared to DB-1, indicating that the overall stiffness of the one-way slabs was effectively enhanced by the fiber mesh fabric. As the load was increased and the bending moment in the flexural zone developed, a progressive reduction in curve slopes was noted between cracking and yielding loads, demonstrating stiffness degradation. The four strengthened slabs demonstrated significantly enhanced load-bearing capacity relative to the control specimen DB-1 at equivalent deflection levels. The specimen CDB-5 exhibits the highest capacity, followed by CDB-4, GDB-3, and GDB-2 in descending order of performance. This enhancement was attributed to the composite action achieved between the fiber mesh and steel reinforcement.

For specimens incorporating the same fiber type but different areal densities, reduced midspan deflections were demonstrated by those with higher-density fiber mesh under identical loading conditions. At a load level of 35 kN, midspan deflections of 6.1 mm and 5.2 mm were recorded for specimens GDB-2 and GDB-3 respectively, while deflections of 4.2 mm and 3.7 mm were measured for specimens CDB-4 and CDB-5. This behavior was explained by the increased content of longitudinal fiber bundles in higher-density meshes, which led to enhanced load-bearing capacity of the mesh system at equivalent tensile strength levels, consequently improving slab stiffness and reducing deflection. When specimens with identical areal densities but different fiber types were compared, smaller midspan deflections were consistently exhibited by carbon fiber mesh reinforced specimens under the same loads. At 50 kN loading, deflections of 15.3 mm and 9.6 mm were observed for specimens GDB-2 and CDB-4 respectively, while 12.7 mm and 8.0 mm were measured for specimens GDB-3 and CDB-5. This performance superiority was derived from the higher elastic modulus and tensile strength characteristics of carbon fiber mesh relative to glass fiber mesh, resulting in increased slab stiffness, improved load-bearing capacity, reduced deflection characteristics, and ultimately higher ultimate load capacities. To further evaluate the energy absorption capacity of the specimens, the flexural toughness was determined by calculating the area under the load-deflection curves up to the defined ultimate state, in accordance with the methodology established in recent relevant literature [41]. The calculated toughness values for specimens DB-1, GDB-2, GDB-3, CDB-4, and CDB-5 were 1252.5 J, 1400.4 J, 1461.0 J, 1268.1 J, and 917.4 J, respectively. The results reveal an important structural behavior influenced by the governing failure criteria. The glass fiber mesh-reinforced slabs (GDB-2 and GDB-3) exhibited the highest energy absorption capacities, as their lower elastic modulus allowed them to sustain high loads while safely deforming up to the 30 mm deflection limit. Conversely, although the high-density carbon fiber mesh (CDB-5) provided the highest ultimate load and stiffness, it reached the strict 1.5 mm crack width limitation at a much smaller deflection (approx. 18.5 mm). This premature termination of the test truncated its post-yield deformation phase, resulting in the lowest calculated ultimate toughness.

### 3.4 Carrying capacity analysis

**Fig 9:**
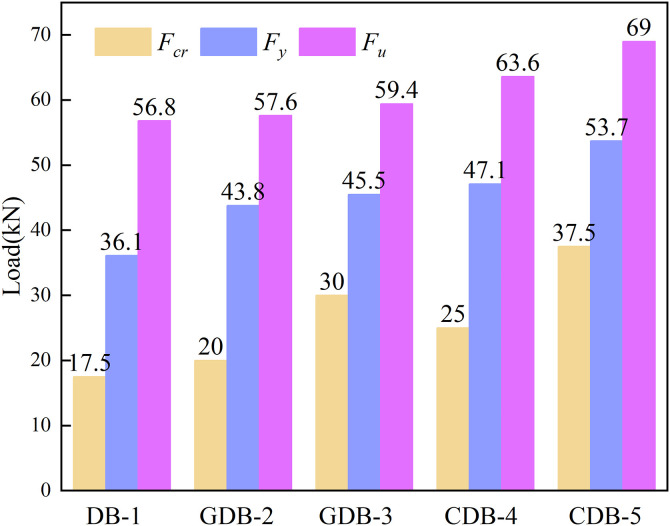
Characteristic load values of the specimens.

Fig 9 presents the experimental values of characteristic parameters including cracking load, yield load, and ultimate load for all specimens. The results clearly demonstrate that the incorporation of fiber mesh fabric significantly enhances the cracking load, yield load, and ultimate load capacity of the specimens. Compared to the control specimen DB-1 without reinforcement, specimens GDB-2, GDB-3, CDB-4, and CDB-5 exhibited percentage increases in cracking load of 14.2%, 71.4%, 42.8%, and 114.2% respectively. The corresponding improvements in yield load were 21.3%, 26.0%, 30.4%, and 48.7%, while the ultimate load capacities increased by 1.4%, 4.5%, 11.9%, and 21.4% respectively. These results confirm that both carbon fiber and glass fiber mesh fabrics effectively improve the characteristic load capacities of the one-way slab specimens.

For specimens with the same fiber type but different areal densities, specimen GDB-3 showed 50%, 3.8%, and 3.1% higher cracking load, yield load, and ultimate load, respectively compared to GDB-2. Similarly, specimen CDB-5 demonstrated percentage increases of 50%, 14.0%, and 8.4% over CDB-4 for these characteristic loads. When comparing specimens with identical areal densities but different fiber types, the carbon fiber-reinforced specimen CDB-5 exhibited 25%, 18.8%, and 16.6% higher cracking load, yield load, and ultimate load respectively than the glass fiber-reinforced specimen GDB-3. Similarly, specimen CDB-4 showed percentage improvements of 25%, 7.5%, and 10.4% over specimen GDB-2 for these key parameters. To provide a more fundamental evaluation of the flexural performance independent of the loading setup, the ultimate bending moments (M_u_) at the mid-span pure bending zone were calculated based on the equilibrium condition M = F × a / 2, where a is the shear span (0.375 m). The calculated ultimate moments for specimens DB-1, GDB-2, GDB-3, CDB-4, and CDB-5 were determined to be 10.65 kN·m, 10.88 kN·m, 11.21 kN·m, 11.93 kN·m, and 12.94 kN·m, respectively. This moment-based comparison perfectly aligns with the load-based analysis, further corroborating that the 240 g/m^2^ carbon fiber mesh integration provides the most substantial flexural enhancement.

### 3.5 Steel reinforcement strain

**Fig 10:**
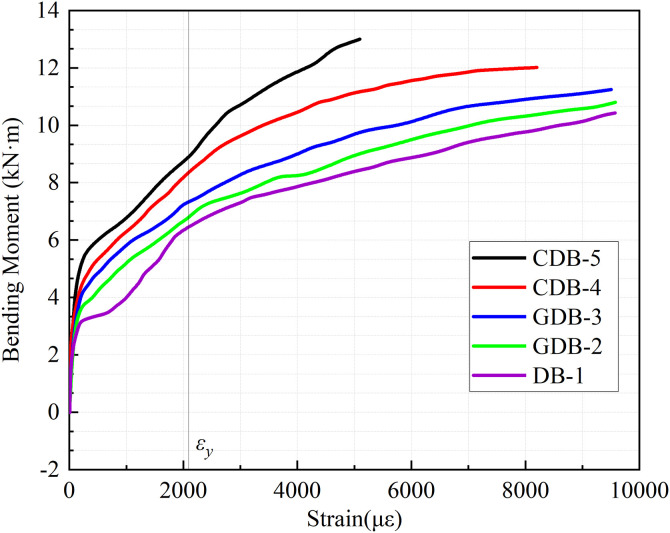
Bending moment-strain curves at mid-span.

Fig 10 presents the bending moment-strain curves of the tensile reinforcement at the midspan bottom location for all specimens. The strain was measured using strain gauges (R3, R4) at the midspan position, and their average value was taken. The curves exhibit similar characteristics across all five one-way slabs, progressing through both elastic and nonlinear growth phases. Under identical loading conditions, the reinforcement strain follows an ascending order from specimen DB-1 to CDB-5, with all specimens ultimately experiencing steel yielding. Prior to reaching 17.5 kN loading, all specimens maintained nearly constant curve slopes, demonstrating a linear relationship between load and reinforcement strain. The control specimen DB-1, lacking fiber reinforcement, displayed a distinct inflection point at 17.5 kN when concrete cracking occurred, resulting in an abrupt strain increase as the load transferred entirely to the reinforcement. In contrast, the reinforced specimens exhibited smoother curves without sharp inflection points due to the composite action between fiber mesh and reinforcement, which enhanced overall stiffness and maintained the concrete’s tensile capacity through non-penetrating cracks.

To move beyond descriptive claims and physically demonstrate this ‘uniform stress distribution’ mechanism, a spatial comparison of the reinforcement strains across the 1/4, midspan, and 3/4 sections was conducted. During the post-cracking to yielding phase, the unreinforced control specimen DB-1 developed a sharp strain gradient; the midspan strain drastically exceeded those at the 1/4 and 3/4 locations due to severe localized stress concentration. Conversely, the fiber mesh-reinforced specimens exhibited a much more gradual and homogenized spatial strain profile. At equivalent load levels, the strain discrepancies between the midspan and the quarter-span locations were significantly reduced. Specimens GDB-2 and GDB-3 with glass fiber reinforcement experienced significant bond failure at loading points, causing a substantial reduction in curve slopes as loads transferred increasingly to the reinforcement. The carbon fiber-reinforced specimens CDB-4 and CDB-5 maintained better composite action due to the superior tensile strength and elastic modulus of carbon fiber mesh, exhibiting more gradual slope reductions.

During the failure stage, distinct strain development patterns were observed among the specimens. The control specimen DB-1 maintained a gradual strain progression, whereas glass fiber-reinforced specimens GDB-2 and GDB-3 displayed rapid strain accumulation caused by sequential fiber mesh failure at loading points. Notably, carbon fiber-reinforced specimens CDB-4 and CDB-5 exhibited the steepest load-strain curves in this phase. This is mainly attributed to the superior stiffness, smaller deflections, and effective stress sharing between the carbon fiber mesh and reinforcement. It is crucial to distinguish demonstrated mechanisms from idealized assumptions. The measured spatial homogenization of strains and finely distributed crack patterns directly prove the fiber mesh’s effective bridging mechanism. However, attributing ultimate failure to a continuous ‘perfect composite action’ remains an idealization. As evidenced by the rapid strain accumulations in glass fiber specimens, this composite action is prematurely disrupted by localized interface debonding well before the fibers’ tensile capacity is fully exhausted.

### 3.6 Concrete strain

**Fig 11:**
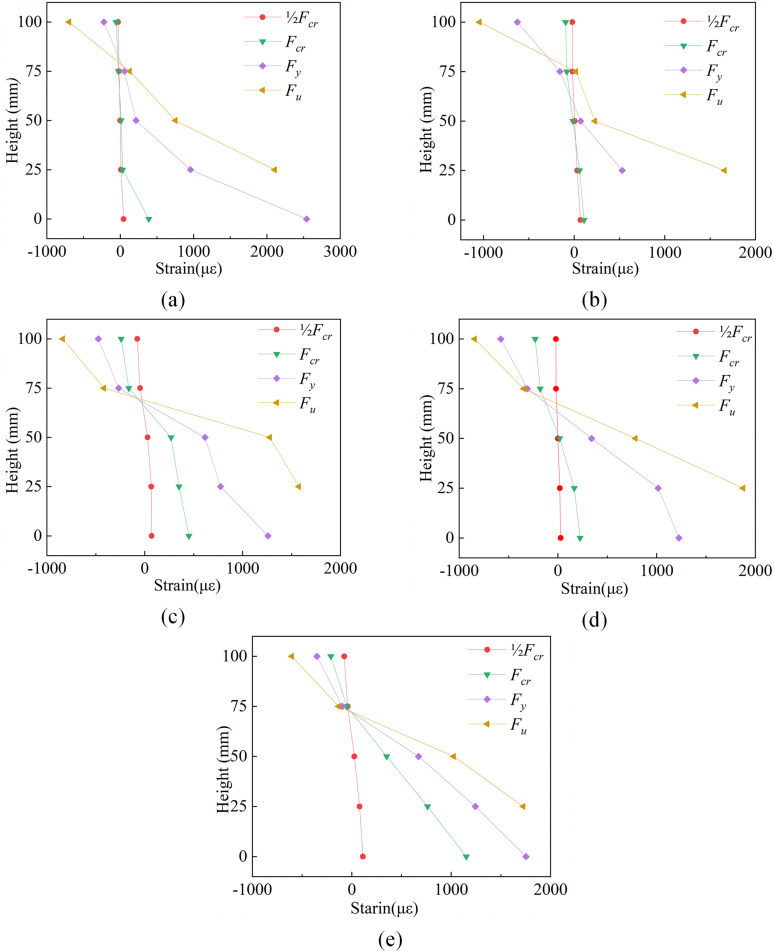
Concrete strain curves. **(a)** DB-1. **(b)** GDB-2. **(c)** GDB-3. **(d)** CDB-4. **(e)** CDB-5.

Fig 11 presents the concrete strain distribution profiles along the cross-sectional height of the specimens, with the bottom surface strain gauge position defined as 0 mm and the top surface corresponding to 100 mm. Before reaching the cracking load, a linear strain distribution through the slab thickness was observed in all specimens, with the neutral axis located approximately at 50 mm height. For the specimen DB-1 without fiber mesh fabric reinforcement, the development of major cracks at midspan was observed during the post-cracking to pre-yielding stage. Significant plastic deformation of the reinforcement caused the concrete strain at the bottom surface to increase sharply from 400 με to 2500 με, ultimately leading to strain gauge failure due to excessive deflection. For specimens GDB-2 and GDB-3 reinforced with glass fiber mesh, slower strain development at the midspan bottom surface was noted, attributed to the deviation of major crack locations from midspan. In contrast, distinct behavior was exhibited by carbon fiber mesh-reinforced specimens CDB-4 and CDB-5. Effective crack bridging by the carbon fiber mesh was demonstrated, maintaining a linear strain distribution at midspan without through-thickness cracking, indicating significant restraint on crack propagation. A comparison of the maximum concrete strains at the ultimate state between DB-1 and FRP mesh-reinforced slabs revealed that all FRP-strengthened specimens exhibited maximum strains below 2000 με, representing a significant reduction compared to the 2500 με maximum strain recorded in DB-1. Furthermore, a progressive reduction in compression zone depth was observed in all specimens. Notably, tensile strains at 75 mm height were detected in specimens DB-1 and GDB-2, suggesting crack propagation beyond the mid-depth of the section. The continuous upward shift of the neutral axis in all specimens confirmed the validity of the plane section assumption for fiber-reinforced concrete one-way slabs, which serves as the foundational basis for analytical sectional equilibrium methods. Furthermore, rather than relying on simplified 1D analytical sectional calculations, these experimental strain distributions and the corresponding cross-sectional equilibrium were rigorously validated through the advanced 3D finite element simulations presented in Section 4.

## 4 Finite element analysis

### 4.1 Modeling method

All the specimens investigated in this study were cast in situ. To account for the interactions among the steel reinforcement, fiber mesh, and concrete, the finite element model was developed under the assumption that no relative slip occurs among these components. Therefore, a discrete modeling approach [42] was adopted for the reinforced concrete slab, which enables a more accurate representation of the structural behavior under external loading, especially in evaluating the localized interaction effects between reinforcement and concrete.

The geometric models of the concrete body, steel reinforcement mesh, and fiber textile were constructed based on the actual specimen dimensions. To prevent numerical convergence issues induced by stress concentrations during loading, rigid bearing blocks were introduced at both the loading and support locations. The concrete and bearing blocks were modeled using three-dimensional, eight-node linear hexahedral elements (C3D8R) [43]. The steel reinforcement was modeled using two-node, three-dimensional truss elements (T3D2).

**Fig 12:**
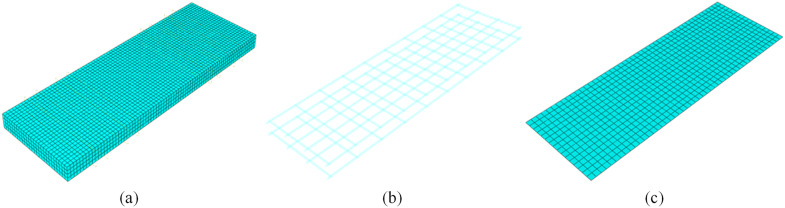
Diagram of grid division. **(a)** Concrete. **(b)** Steel reinforcement. **(c)** Fiber mesh fabric.

The fiber mesh, which exhibits anisotropic mechanical properties in the longitudinal and transverse directions, was represented with three-dimensional shell elements (S8R), as shown in Fig 12. To justify the representation of the open grid as a continuous sheet without artificially inflating the structural stiffness, an equivalent membrane stiffness approach was employed [44]. Specifically, the equivalent thickness ($t_{eq}$) of the shell elements was calculated by smearing the cross-sectional area of the individual fiber bundles ($A_{f}$) over the corresponding grid spacing (s), formulated as $t_{eq}=A_{f}/s$. This ensures the membrane stiffness in the principal directions remains equivalent to the physical grid.

### 4.2 Boundary conditions

The interaction between the reinforcement and the concrete was defined using the “Embedded” constraint, while the fiber textile was bonded to the bottom surface of the concrete slab using the “Tie” constraint. A surface-to-surface contact interaction was defined between the bottom surface of the concrete slab and the rigid bearing blocks, with a tangential friction coefficient of 0.01 and “Hard” contact behavior in the normal direction. The loading point was tied to the top surface of the concrete, and a reference point was established and coupled with the loading surface to apply the load.

**Fig 13:**
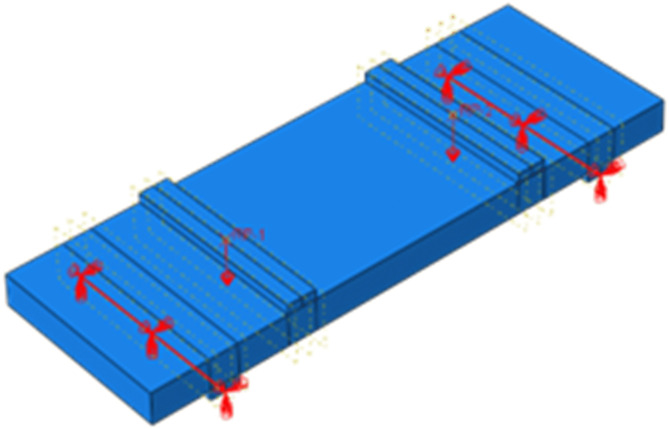
Boundary conditions.

The slab was modeled under simply supported conditions. One end was assigned a sliding hinge support, while the other end was given a fixed hinge support. To replicate the actual support conditions and allow the rigid bearing blocks to rotate with the slab, appropriate boundary constraints were applied along the central axis of the support blocks. The sliding hinge support restrained displacements in the vertical (U2) and transverse (U3) directions, as well as rotations about the longitudinal (UR1) and transverse (UR3) axes (i.e., U2 = U3 = UR1 = UR3 = 0). The fixed hinge support restrained all translational and rotational degrees of freedom except for rotation about the vertical axis (i.e., U1 = U2 = U3 = UR1 = UR3 = 0). A concentrated force was applied at the loading point, as illustrated in Fig 13.

### 4.3 Material constitutive models

The constitutive behavior of concrete was modeled using the Concrete Damage Plasticity (CDP) model available in ABAQUS, following the approach described in [45]. This model is well-suited for simulating damage evolution and crack propagation under monotonic, cyclic, and dynamic loading conditions. It assumes that the concrete is a continuous and isotropic material, exhibiting tensile cracking under tension and crushing under compression. The degradation of elastic stiffness is governed by damage parameters, which can effectively capture the onset and progression of cracks.

The input parameters for concrete included an elastic modulus *E*_*c*_ = 3.08 × 10^4^ MPa and a Poisson’s ratio 𝜈_*c*_ equal to 0.2. The tensile and compressive damage factors were defined using Equations (2) and (3), where the damage coefficients for tension and compression, *b*_*t*_ and *b*_*c*_, were taken as 0.1 and 0.7, respectively. The basic parameters used in the CDP model are summarized in Table 5.

**Table 5:** Basic parameters of CDP.

Expansion angle	Eccentricity	*f*_b0_/*f*_c0_	*K*	Viscosity parameter
30	0.1	1.16	0.667	0.0001


$$\begin{array}{c} d_{c}=1-\frac{\sigma_{c}E^{-1}}{\varepsilon_{c}(1/b_{c}-1)+\sigma_{c}E^{-1}} \end{array}$$ (2)

$$\begin{array}{c} d_{t}=1-\frac{\sigma_{t}E^{-1}}{\varepsilon_{t}(1/b_{t}-1)+\sigma_{t}E^{-1}} \end{array}$$ (3)

The steel reinforcement bars were modeled using an ideal elastic-plastic material with linear hardening, as described in [46]. The input parameters included an elastic modulus *E*_*s*_ = 200GPa and Poisson’s ratio ν_*s*_ equal to 0.3. For the fiber mesh grid material, an orthotropic linear elastic constitutive model was used, based on the assumption that fiber bundles fail by rupture once the tensile strength is exceeded. Based on the equivalent membrane stiffness principle and the material properties obtained from tensile tests (Table 3), the equivalent thicknesses and orthotropic elastic moduli ($E_{1}$ and $E_{2}$)for the longitudinal and transverse directions, respectively) were explicitly defined in the ABAQUS material module. The elastic modulus of the glass fiber mesh was set as $E_{f}$ =68 GPa, while that of the carbon fiber mesh was 198 GPa in their primary load-bearing directions, with a drastically reduced out-of-plane stiffness to simulate the negligible bending resistance of the textile. A Poisson’s ratio $v_{f}$ of 0.28 was used for both fiber types.

### 4.4 Comparative analysis of numerical and experimental results

#### 4.4.1 Load-deflection curves.

**Fig 14:**
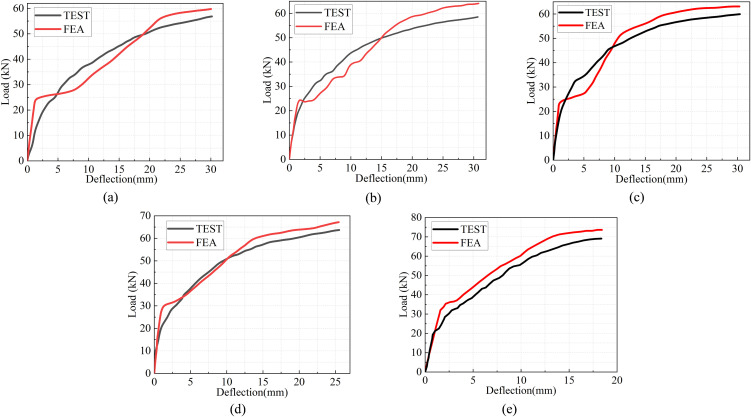
Experimental and simulated load–deflection curves. **(a)** DB-1. **(b)** GDB-2. **(c)** GDB-3. **(d)** CDB-4. **(e)** CDB-5.

Fig 14 compares the experimental and simulated load-deflection curves for all slab specimens. For the slab DB-1. The numerical simulation predicted a higher initial stiffness during the elastic stage than the experimental results. The curve exhibited a distinct displacement jump at the onset of concrete cracking, followed by a smooth transition period as the tensile failure of the bottom concrete developed and the reinforcement progressively carried the bending moment, reflecting the cracked section behavior. A second displacement discontinuity appeared at the steel yielding point, accompanied by a sharp reduction in the curve slope. The simulation overestimated the ultimate load capacity, which can be attributed to idealized modeling assumptions. These include perfect bond between reinforcement and concrete without consideration of bond-slip effects, and the adoption of a linear hardening elastoplastic model for the steel reinforcement.

The glass fiber mesh fabric-reinforced slabs (GDB-2 and GDB-3) and carbon fiber mesh fabric-reinforced slabs (CDB-4 and CDB-5), depicted in Fig 14(b) through 14(e), were modeled with the fiber mesh fabric as shell elements perfectly bonded to the concrete bottom surface. All fiber-reinforced specimens demonstrated greater initial stiffness in simulations compared to experiments. This discrepancy was primarily due to the shell elements failing to fully replicate the actual grid characteristics of the textile mesh, instead idealizing it as an elastic material embedded in the concrete matrix. The glass fiber mesh models experienced failure at certain load levels due to their relatively low elastic modulus and tensile strength. In contrast, the carbon fiber mesh models showed better agreement with experimental curves, owing to their superior mechanical properties. Similar to DB-1, all simulations predicted higher ultimate loads than the corresponding experimental results because the models did not account for bond-slip behavior between reinforcement, fiber mesh, and concrete.

Table 6 summarizes the ultimate load values and error rates for the specimens. The differences between simulated and experimental ultimate loads were 5.1%, 9.8%, 6.8%, 5.6%, and 6.5%, respectively. These deviations mainly resulted from idealized assumptions, including the neglect of bond-slip effects and simplified constitutive relationships. Despite these simplifications, the finite element models captured the overall structural behavior well, though stiffness and load capacity were slightly overestimated. This overestimation is inherently attributed to the idealized ‘Tie’ and ‘Embedded’ constraints utilized in the model, which assume a perfect bond and neglect the interface slip and localized debonding observed in the experiments. Therefore, rather than serving as an exact quantitative validation, the current FE results should be repositioned and interpreted as a qualitative tool. The numerical models establish an idealized upper-bound representation of the global flexural performance assuming perfect composite action, while the actual crack control capabilities are inevitably lower due to interface damage.

**Table 6:** Comparison of the ultimate loads for experimental and simulated results.

Specimen	Data type	Ultimate load (kN)	Error rate %
DB-1	TEST	56.8	5.1
	FEA	59.7	
GDB-2	TEST	58.0	9.8
	FEA	63.7	
GDB-3	TEST	59.8	6.8
	FEA	63.9	
CDB-4	TEST	63.6	5.6
	FEA	67.2	
CDB-5	TEST	69	6.5
	FEA	73.5	

#### 4.4.2 Concrete damage.

**Fig 15:**
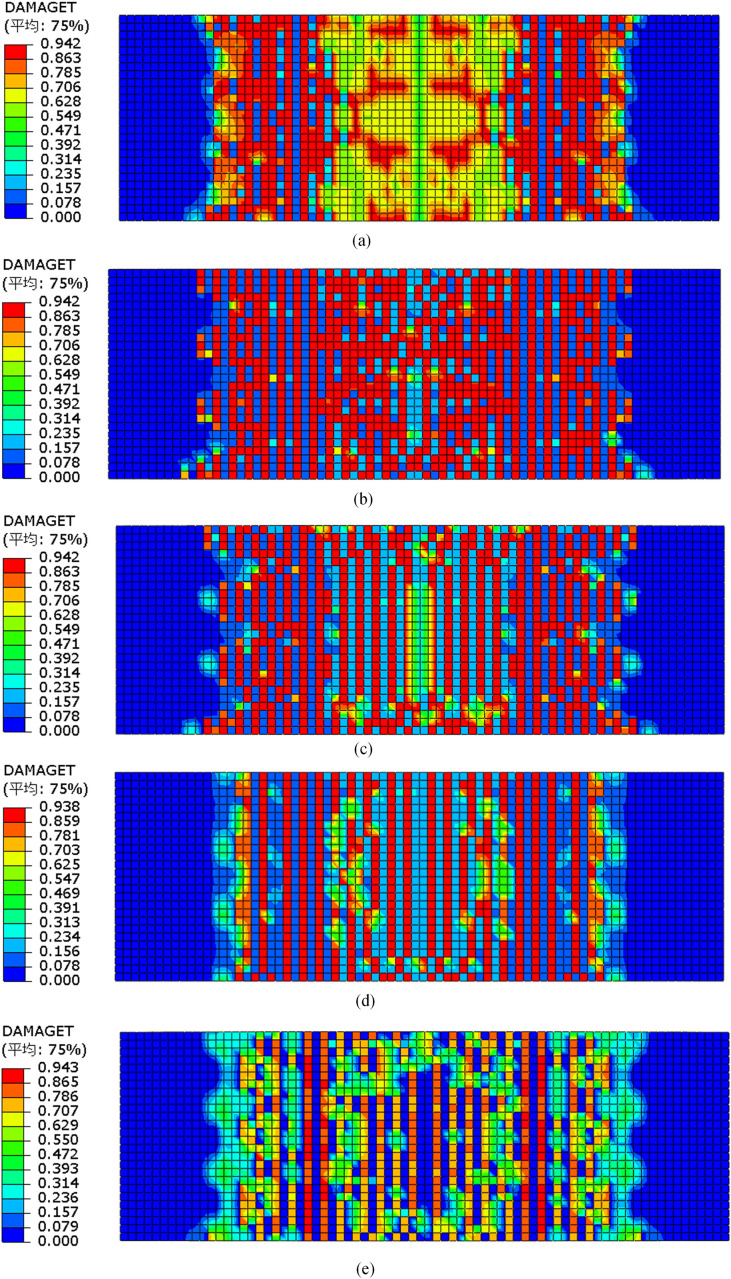
Cloud map of concrete tensile‌‌ damage. **(a)** DB-1. **(b)** GDB-2. **(c)** GDB-3. **(d)** CDB-4. **(e)** CDB-5.

Fig 15 presents the tensile damage contour plots of each specimen at failure. The results indicate that tensile damage in the bottom concrete primarily occurred within the pure bending region, exhibiting typical flexural failure characteristics. In the finite element model, concrete was assumed to behave as an isotropic material. A tensile damage factor of 0.9 was taken to indicate the onset of cracking. However, the contour plots generated from the numerical model cannot accurately reproduce the precise crack shapes and widths observed in the experiments. Instead, they identify regions of high damage concentration, and the approximate crack locations must be inferred from the distribution of these damaged elements.

For the slab DB-1, notable damage appeared near the loading points and support regions, with damage factors ranging from 0.628 to 0.785 across most of the span. In contrast, GFRP-reinforced specimens GDB-2 and GDB-3, as well as CFRP-reinforced specimen CDB-5, exhibited damage values reaching 0.9 within the mid-span region, suggesting significant cracking. For specimen CDB-4, however, such high damage was only observed near the loading points, indicating that crack development in the span region was effectively suppressed. Overall, the predicted damage patterns from the simulations showed good agreement with the experimentally observed cracking behavior. Moreover, the damage distributions provide further insight into the influence of different types of fiber reinforcement on failure mechanisms. The unreinforced specimen DB-1 exhibited widespread damage propagation, while the fiber-reinforced slabs showed more localized damage. Compared to GFRP, CFRP mesh offers higher elastic modulus and tensile strength, leading to better reinforcement performance. In particular, specimen CDB-4 demonstrated the effectiveness of high-density CFRP mesh in restricting crack propagation toward the mid-span region, concentrating damage around the loading points and thereby improving crack resistance and structural stability.

**Fig 16:**
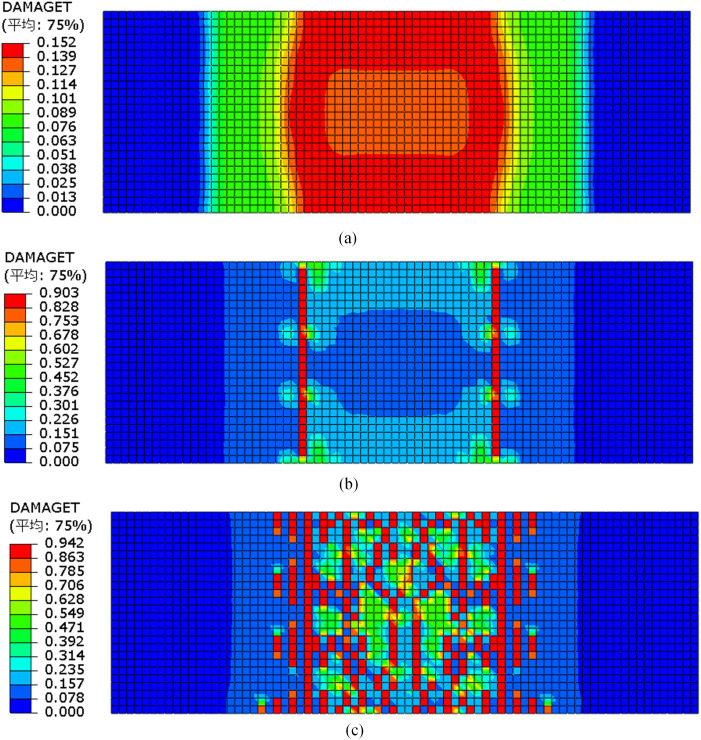
Tensile damage cloud map for concrete at different stages for GDB-2. **(a)** 15kN before cracking. **(b)** 20kN when cracking. **(c)** 43kN when yielding.

Due to the similar failure processes observed among the fiber mesh fabric-reinforced specimens, specimen GDB-2 was selected as an example to illustrate the evolution of tensile damage in the bottom concrete surface at three critical loading stages. Fig 16 presents the tensile damage contours at 15 kN (pre-cracking stage), 20 kN (crack initiation), and 43.8 kN (steel yielding stage).

At the 15 kN loading stage, the bending moment in the pure flexural zone gradually increased, leading to the continuous accumulation of tensile stress in the concrete, approaching its tensile strength. When the load reached 20 kN, localized damage first appeared near the loading points, indicating the onset of cracking. As the load increased to 43.8 kN, corresponding to the yield point of the reinforcement, the damaged region expanded significantly in the mid-span area, accompanied by the formation and development of multiple cracks. With further loading, the damage continued to propagate toward both support regions. The finite element simulation accurately captured the characteristic sequence of crack development observed during the experiment.

#### 4.4.3 Steel reinforcement stress.

**Fig 17:**
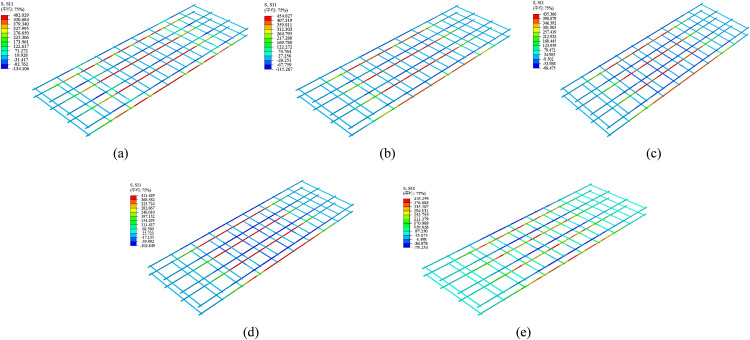
Stress cloud map for steel reinforcement. **(a)** DB-1. **(b)** GDB-2. **(c)** GDB-3. **(d)** CDB-4. **(e)** CDB-5.

Fig 17 displays the steel reinforcement stress contours at the ultimate loading stage for all specimens. The results indicate that compressive stresses developed in the top reinforcement, while the bottom longitudinal reinforcement experienced tensile stresses exceeding 418 MPa, signifying yielding. For all five specimens, the peak tensile stress occurred in the pure bending zone near the midspan and gradually diminished toward the supports. The specimen DB-1 exhibited the highest reinforcement stress, reaching a maximum of 482 MPa. In comparison, the fiber mesh fabric-reinforced specimens GDB-2, GDB-3, CDB-4, and CDB-5 demonstrated slightly reduced stress levels in the reinforcement. This reduction is attributed to the effective distribution of tensile forces between the steel reinforcement bars and the fiber mesh reinforcement, which provided additional tensile capacity. These numerical results align well with the experimental findings.

### 4.5 Limitations of the study

Due to experimental conditions and logistical constraints, the results presented in this study primarily reflect nominal performance improvements under specific structural configurations and cannot be statistically generalized. The idealized perfect bond assumption used in the finite element analysis did not account for the interface slip phenomena that were present in the experiments. Furthermore, localized debonding and cover spalling led to the early limitation of the strengthening efficiency, indicating that future research should introduce more realistic interface behavior descriptions into numerical models and reinforcement system designs. This could involve integrating discrete bond-slip models, interface damage evolution, and mechanical anchorage effects to more accurately reflect the force mechanisms and failure processes of the mesh reinforcement system [47,48].

## 5 Conclusion

This study comprehensively evaluated the flexural behavior of reinforced concrete (RC) one-way slabs incorporating embedded carbon and glass fiber mesh fabrics through experimental testing and finite element simulations. Based on the results, the following conclusions can be drawn:

(1) All specimens exhibited typical flexural failure modes, with multiple cracks initiating between the loading points. The incorporation of fiber mesh fabrics significantly improved crack control by limiting crack widths and preventing through-cracks. Notably, the 240 g/m^2^ carbon fiber mesh fabric exhibited the most effective crack suppression, producing the narrowest crack zone and the lowest crack density.
(2) Under the specific conditions of this single-specimen experimental program, the fiber mesh fabrics considerably enhanced the structural performance of the RC slabs. Compared to the unreinforced control specimen, the cracking load exhibited a nominal increase of 14.2%–114.2%, the yield load by 21.3%–48.7%, and the ultimate load by 1.4%–21.4%. Among all tested specimens, the slab reinforced with 240 g/m^2^ carbon fiber mesh achieved the highest nominal improvement across all loading stages.
(3) Before initial cracking, all specimens showed similar strain development trends. After cracking, slabs reinforced with fiber mesh fabrics exhibited lower reinforcement and concrete strains under the same applied loads compared to the control, indicating effective stress redistribution and delayed reinforcement yielding due to the fiber-concrete composite action.
(4) The finite element analysis accurately reproduced the observed crack patterns and damage development, particularly at the slab’s tensile zone. The simulations confirmed the yielding of steel reinforcement and highlighted the synergistic interaction between steel bars, fiber mesh, and concrete. This interaction contributed to enhanced post-cracking performance, improved load-sharing behavior, and overall structural stability.

## Supporting information

S1 File Source data. (XLSX)
